# Research on path planning for clear aligner flexible manufacturing

**DOI:** 10.1038/s41598-024-64546-y

**Published:** 2024-07-01

**Authors:** Kuntao Huang, Sheng Gao, Cheng Zou, Cong Zhang, Yili Peng, Xi Liu

**Affiliations:** 1 School of Mechanical & Electrical Engineering, Wuhan Institute of Technology, Wuhan, 430205 China; 2 Hubei Research Center of Intelligent Welding Equipment and Software Engineering Technology, Wuhan Institute of Technology, Wuhan, 430205 China; 3 State Key Laboratory of Intelligent Manufacturing Equipment and Technology, Wuhan, 430223 China; 4 Suzhou Hyperion Robotics Technology Co., Ltd, Suzhou, 215299 China

**Keywords:** PCA-ICP registration, Clear aligner manufacturing, Robot milling path planning, Information technology, Mechanical engineering

## Abstract

In recent years, clear aligner can enhance individual appearance with dental defects, so it used more and more widely. However, in manufacturing process, there are still some problems, such as low degree of automation and high equipment cost. The problem of coordinate system mismatch between gingival curve point cloud and dental CAD model is faced to. The PCA-ICP registration algorithm is proposed, which includes coarse match algorithm and improve-ICP registration algorithm. The principal component analysis (PCA) based method can roughly find the posture relationship between the two point clouds. Using z-level dynamic hierarchical, the ICP registration can accurately find the posture between these two clouds. The final registration maximum distance error is 0.03 mm, which is smaller than robot machining error. Secondly, the clear aligner machining process is conducted to verify the registration effectiveness. Before machining, the path is generated based on the well registered gingival curve. After full registration, the tool path is calculated by establishing a local coordinate system between the workpiece and the tool to avoid interference. This path is calculated and generated as an executable program for ABB industrial robots. Finally, the robot was used for flexible cutting of clear aligners and was able to extract products, ensuring the effectiveness of the proposed research. This method can effectively solve the limitations of traditional milling path planning under such complex conditions.

## Introduction

### Background

In recent years, with the improvement of people’s living standards, there has been an increasing emphasis on personal appearance. Having a set of clean and well-aligned teeth is crucial for enhancing one’s self-image. Handicapping dentofacial anomalies can affect dento-maxillofacial growth, oral function, oral health, and also have an impact on one’s self-appearance. This will cause psychological distress^1,2^. Nowadays, the treatment for dental malformations involves wearing orthodontic appliances.

**Figure 1:**
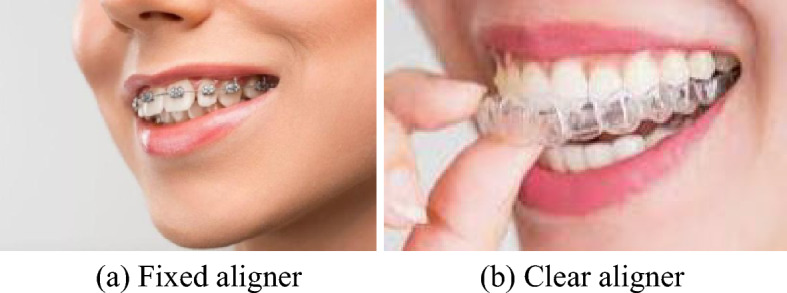
Two types of aligners.

There are mainly two types of orthodontic aligners, that is, fixed aligner and clear aligner. Fixed aligner, as shown in Fig. 1a, are the most commonly used type due to their simple structure, mature manufacturing technology, and lower cost. Figure 1a is the typical fixed aligner, while square wire aligner is the most widely used. Figure 1b is clear aligner, which offers better aesthetic effects, higher comfort, and shorter treatment duration. For complex orthodontic treatments, fixed aligners are more suitable^3^, while clear aligners are obviously more appropriate for cases where malocclusion is not particularly severe^4^.

Due to its aesthetic appeal and increased comfort, the use of clear aligners has become more widespread in daily clinical practice. Such an orthodontic method involves wearing a series of clear aligners that have gradually shape change in order to progressively move the teeth to suitable positions. Each aligner will gradually reposition one or more teeth a small distance. Clear aligners were introduced in the 1950s^5^ and were approved for production and use by the US Food and Drug Administration in 1998^6^. Primary orthodontic treatments were planned and manufactured based on manual operations by professionals. This specific limitation restricted the widespread use of orthodontic aligners.

In recent years, the rapid development of computer-aided design (CAD) and rapid prototyping (RP) technologies has enabled fast planning of orthodontic treatments. The manufacturing of polymer orthodontic appliances based on thermoplastic materials can now be done using industrial methods for mass production.

Currently, the processing steps of orthodontic appliances include several stages: digitization of dental models; digital simulation of treatment planning; additive manufacturing of the master model; thermoforming technology for aligner fabrication; and cutting of the membranes to take out the aligner. Five steps are shown in detail^7^.

- Step 1: Non-contact 3D model scanning devices are used to scan the teeth of the patient, obtaining a digital model of the oral cavity.
- Step 2: Orthodontists use dental software to develop a treatment plan, while orthodontic technicians plan out the movement of each tooth and create a series of treatment dental models.
- Step 3: 3D printing is utilized to create physical dental models.
- Step 4: Thermoforming is used to press the membranes onto the dental models, resulting in formed orthodontic aligner membranes.
- Step 5: The membranes are cut using computer numerical control (CNC) machining to take out the orthodontic aligner, resulting in the final product.

Although the current orthodontic treatment plans are relatively mature, and there are commercially available dental model processing software, there is still limited research on the digital manufacturing of orthodontic aligner. Currently, the commonly used method for cutting orthodontic appliances is manual cutting. Most of the software available on the market for orthodontic appliances focuses on treatment planning^8^, but lacks CAM software related to appliance cutting. This paper focuses on the research work of digital manufacturing of orthodontic aligner.

### Literature review

The development of computer-aided design (CAD) and rapid prototyping (RP) techniques has allowed an industrial approach for both planning orthodontic treatments and manufacturing polymeric aligners^7^. Digital reconstructions of dental crowns are conducted by optically scanning either a plaster model or the patient's mouth^9^.

Dental crowns digital reconstructions can finish by 3D scan technology. However, to plan an accurate orthodontic treatment is still a challenge job. The teeth movement is one of skilled problem. In orthodontic treatment, some teeth should be designed specified movement, and auxiliary elements should be added on the aligner. Orthodontic softwares, such as 3Shape, SmartForce, have automatic teeth segmentation function and teeth movement function^10,11^. Artificial intelligence algorithm finds the gingival border of each tooth. This is the critical algorithm for the subsequent steps and it is the secret of commercial software companies. However, these softwares are complicated and users cannot easily learn and master them in a short period of time for diagnosis and treatment. Additionally, in orthodontic treatment process, how to control teeth movement and how to evaluate the mechanical performance of tooth movement still requires professional orthodontist to handle. Ref^12^ researches on how clear aligner delivers forces and moments to tooth surface. The rule of forces and moments will allow the design to more efficient orthodontic treatment. In addition, Tarek M. Elshazly^13^ study the effect of the geometry and the extension of orthodontic aligner edges and the aligner thickness on force transmission to upper right central incisor tooth. The design of the trimming line, the edge extension, and the thickness of the aligner affect significantly the magnitude of the resultant force and the distribution of normal contact force. The straight extended trimming design exhibited better force distribution that may favor a bodily tooth movement. The manufacturing procedures of the straight design are much simpler compared to the scalloped design. Ref^14^ introduces a computational design and engineering framework, which allows patient-specific simulations of mechanical interactions between dental tissues. The framework parametrics modeling of aligner shapes and use finite element modeling of tooth-aligner interactions. In clear aligner orthodontics, the use of attachments in conjunction with aligners can better control tooth movement. Ahmad^15^ conducted a study using three-dimensional finite element analysis to determine the biomechanical effects of attachment geometry on orthodontic forces and moments. By selecting appropriate attachment sizes, appropriate forces and moments required for specific clinical patients can be obtained.

The manufacturing process of invisible aligners involves 3D printing of dental models, followed by cutting the film sheets pressed onto the models to form the final aligner product. The film sheets are removed from the dental models and cut manually using scissors. After cutting, the sharp edges are removed by polishing^8^. Alternatively, there is a solution available in the market, like iNLASE®^16^, where laser cutting is used to cut the film sheets. Laser cutting, being a continuous process, prevents the formation of sharp edges.

Due to the complex shape of dental models, both cutting and laser processing for aligner manufacturing typically require the use of multi-axis machining techniques to achieve accurate path planning and cutting. The key issues in multi-axis machining can be divided into two aspects, gouge and collision avoidance and smoothness.

In recent years, the application of equipping robots with electric spindles at their end effectors to enable machining capabilities has been widely researched and implemented^17–19^. Compared to machine tool machining, robot machining has several advantages, including intelligence, high collaboration, low cost, good flexibility, large workspace, and good spatial accessibility.

The rest of this paper is organized as follows. We first introduce the problem to solve in this paper and the whole process in Section "Statement description". We then present our proposed improved registration algorithm based on PCA-ICP in Section "Improved registration algorithm based on PCA-ICP". The algorithm will be validated and discussed in Section "Algorithm validation and processing experiment". Finally, we conclude this paper in Section "Finally, according to Eq. (7), the homogeneous transformation matrices and corresponding to the two point clouds are calculated.".

## Statement description

### Problem description

**Figure 2:**
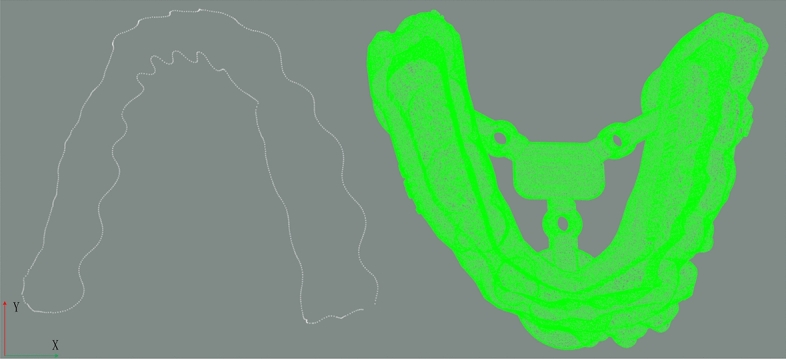
Coordinate system mismatch.

The common practice of generating gingival curves in the orthodontic software is as below. The oral scanner is used to scan the oral cavity to obtain dental CAD model point cloud. When the point cloud is imported into the orthodonic software, like 3-Shape, the software provides the function that recognize a curve or drawing a closed curve on this model, based on the orthodontist treatment strategy and customer wearing habit. This curve is called gingival curve in this paper. The dental CAD model and generated gingival curve is built from the oral scanner. If it is imported in the machining environment, it is necessary to find a datum to fix the dental model. The oral scanner and the software may try to find the datum, but due to the tooth complexity and diversity, not all datums are suitable for fixing and finish the clear aligner machining. So the first problem is to match the model in oral scanner and machining environment. In the machining process, the gingival curve is decided by orthodontist, while the dental CAD model is fixed in machining process. Maybe align the dental CAD models in oral scanner and machining environment is convenient in registration algorithm, but from the side by clear aligner manufacturer, it is not convenient to provide the two dental CAD models. So the dental model in oral scanner is missing. The difficulty to solve the aligning problem increased. The aligning problem becomes to align the gingival curve to the dental CAD model, as shown in Fig. 2. So this paper is trying to solve this first problem.

The second problem is that when gingival curve and CAD model are both provided, the machining path generation is not easy. The shape of dental model is compact and steep. The robot moving along this curve cannot be smooth. If the orientation sequence is not optimized, the machining motion will be with very large acceleration that leads to a very low efficiency. So how to optimize the robot moving is the robot cutting path planning problem.

### Algorithm guideline

**Figure 3:**
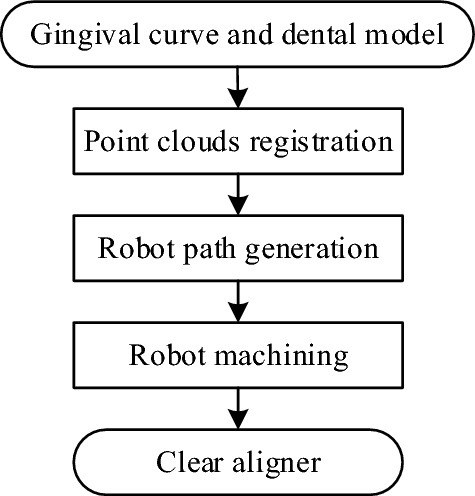
The whole clear aligner manufacturing flowchart.

The whole solution is divided by 3 main steps. The input datas are gingival curve and dental model data obtained from orthodontic software and machining device. As shown in Fig. 2, the additional part with three holes is printed with the dental model and this part is used for fixed in the machining environment. In this paper, the tooth is fixed on the robot. The first main step is registration and alignment of the gingival curve point cloud and the dental model point cloud. The second step is robotics milling path planning based on the registered gingival curve. In the last, the clear aligner is milled by the industrial robot. The whole flow path is in Fig. 3.

### Ethical approval

The authors declare that there are no conflicts with the ethical standards by Springer and the research conducted in this research paper.

### Consent to participate

Consent was obtained from all individuals included in the study.

## Improved registration algorithm based on PCA-ICP

### Overview

The ICP algorithm is the most commonly used point cloud registration algorithm. However, the algorithm has poor robustness to point clouds with noise and missing data, and it is often difficult for complete registration. The initial pose of the point cloud to be registered is required to be relatively precise; otherwise it will fall into local optimal solution. In view of the above problems and combined with the actual data, the gingival curve point cloud is a three-dimensional point sequence without surface features, which can not extract accurate feature information, such as normal, curvature, etc. At the same time, because the gingival curve point cloud data is directly selected on the dental model, some similar geometric information is retained between them.

**Figure 4:**
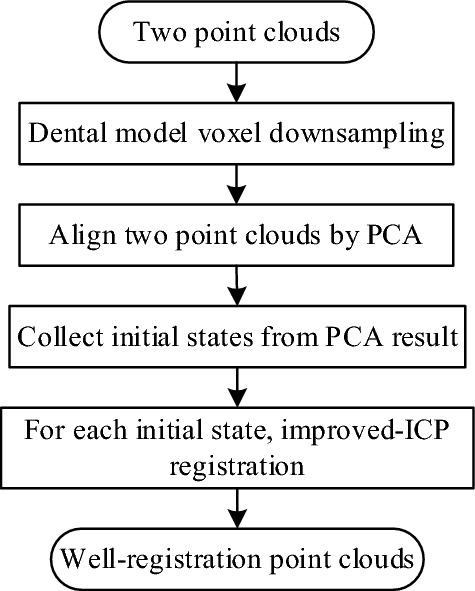
The overall flow chart of the algorithm.

Therefore, in the coarse registration stage, the PCA principal component analysis method is used for the two sets of point clouds, and then the Euclidean distance between the point clouds is used as the input parameter of the orientation dynamic adjustment mechanism, and a method of combining dynamic matching point pairs with ICP coarse registration is also adopted to solve the disadvantages of some principal component analysis methods. Finally, in order to effectively solve the problem that the traditional ICP algorithm is easy to fall into the local optimal solution, this Section proposes an improved dynamic ICP algorithm to complete the fine registration. The details of the registration process are in Fig. 4.

### PCA-based coarse registration algorithm

**Figure 5:**
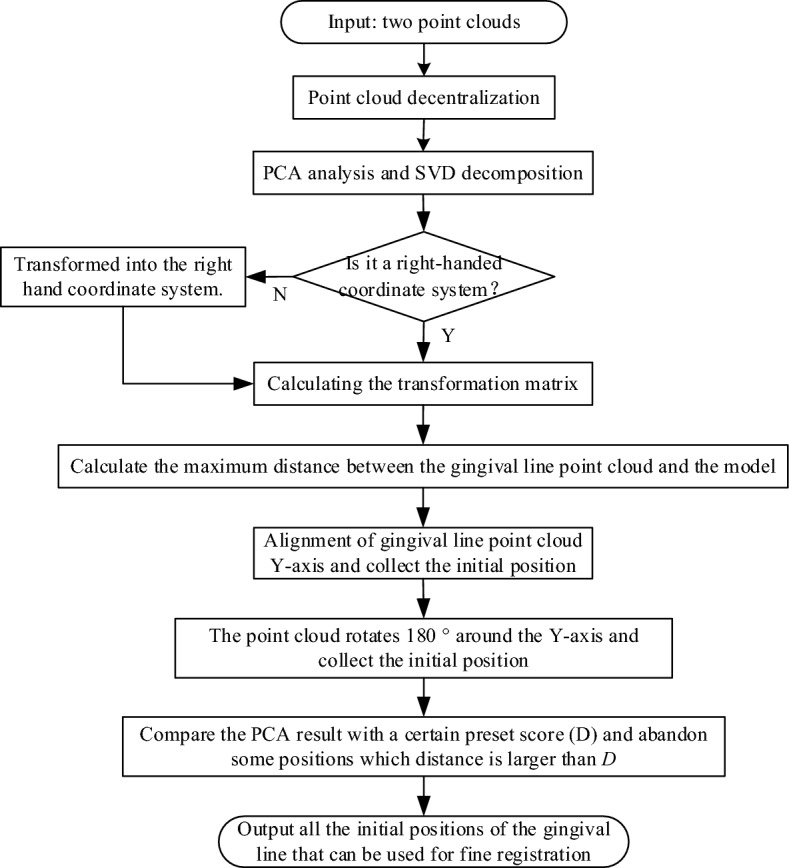
An improved PCA coarse registration flow chart.

This paper proposes an improved coarse registration algorithm. The details of the registration process are shown in Fig. 5. If directly using the PCA method, the principal axis of two point clouds can be aligned, but the coordinate system may be reversed. So an improved coarse registration algorithm is proposed based on PCA.

#### Principal component analysis (PCA) method

PCA is a widely used algorithm for dimensionality reduction, which can reduce the dimensionality of data while retaining key information. This article adopts the PCA algorithm based on SVD singular value decomposition for coarse registration. The specific steps are as follows:

1. For two point cloud *A* and *B*, the covariance matrix of point cloud can be obtained as Eq. (1) and (2) by decentralization.
  1 $$C_{A} = \frac{1}{N - 1}\mathop \sum \limits_{i}^{N} \left( {a_{i} - \overline{a}} \right)\left( {a_{i} - \overline{a}} \right)^{T} , \overline{a} = \frac{1}{N}\mathop \sum \limits_{i = 1}^{N} a_{i}$$
  2 $$C_{B} = \frac{1}{M - 1}\mathop \sum \limits_{j}^{M} \left( {b_{j} - \overline{b}} \right)\left( {b_{j} - \overline{b}} \right)^{T} ,\overline{b} = \frac{1}{M}\mathop \sum \limits_{j = 1}^{M} b_{j}$$
  *a*_i_ is the *i*th point of point cloud *A*, the same as *b*_i_ and *B*.
2. The covariance matrices $C_{A}$ and $C_{B}$ are decomposed by SVD.
  3 $$C_{A} = U_{1} S_{1} V_{1}^{T} C_{B} = U_{2} S_{2} V_{2}^{T}$$
  Here, to transform the point cloud to basic coordinate system, the rotation matrix can be written as *R*_A_ and *R*_B_.
  4 $$R_{A} = V_{1} U_{1}^{T} R_{B} = V_{2} U_{2}^{T}$$
3. For each point, the aligned point clouds *A’* and *B’* are calculated.
  5 $$a^{\prime} = \left( {R_{A} } \right)a + \overline{a}$$
  6 $$b^{\prime} = \left( {R_{B} } \right)b + \overline{b}$$
  The transformation matrix from original to aligned point clouds is *T*_A_ and *T*_B_.
  7 $$T_{A} = \left[ {\begin{array}{*{20}c} {R_{A} } & {\overline{a}} \\ 0 & 1 \\ \end{array} } \right] T_{B} = \left[ {\begin{array}{*{20}c} {R_{B} } & b \\ 0 & 1 \\ \end{array} } \right]$$
4. The gingival curve point cloud is used as the point cloud *A* that needs to be matched and the dental model point cloud is used as the target point cloud *B*. The two point clouds are processed by the traditional PCA principal component analysis algorithm. To avoid the different situations of the left-handed coordinate system and the right-handed coordinate system of the two point cloud local coordinate systems, resulting in the occurrence of the z-axis reverse situation, so it is adjusted to a unified right-handed coordinate system. The adjusting method is as follow. Calculate the covariance matrices $C_{A}$ and $C_{B}$ according to Eq. (1) and Eq. (2), calculate $U_{1}$ and $U_{2}$ using Eq. (3), and then the first column and the second column vectors are crossed and multiplied to obtain vectors $V_{1} \left( {x1,y1,z1} \right)$ and $V_{2} \left( {x2,y2,z2} \right)$. The third column vector of $U_{1}$ is updated by vector $V_{1}$, and the third column vector of $U_{2}$ is updated by vector $V_{2}$.
5. Finally, according to Eq. (7), the homogeneous transformation matrices $T_{A}$ and $T_{B}$ corresponding to the two point clouds are calculated.

**Figure 6:**
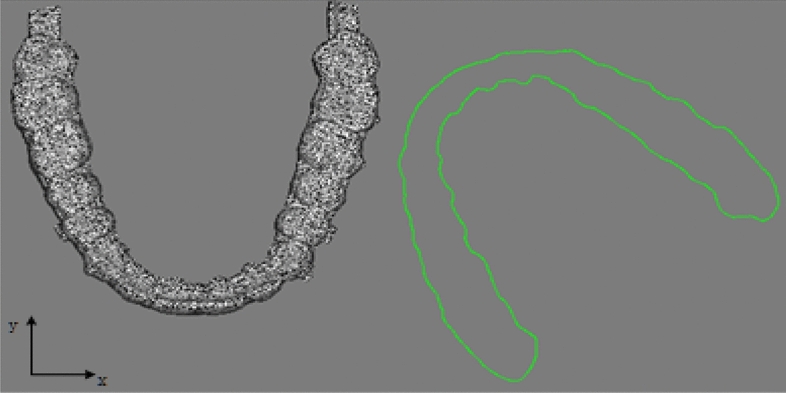
Initial pose.

**Figure 7:**
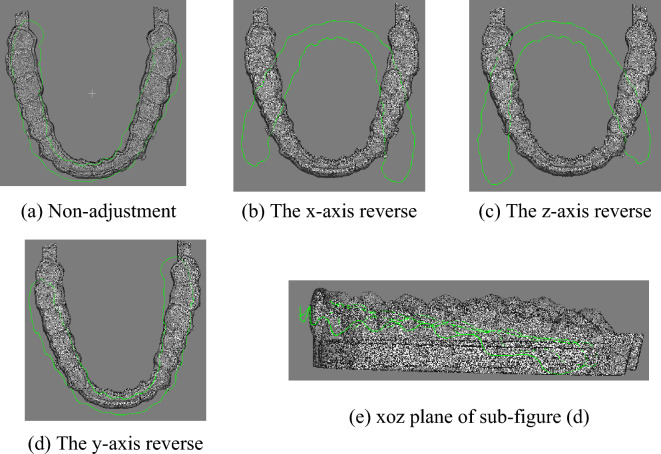
All possible results of the PCA algorithm.

As shown in Figs. 6 and 7, all possible results after traditional PCA analysis and adjusted coordinate system alignment were performed on the two point clouds. In particular, it should be noted that all cases of axis alignment or reverse mentioned in this paper are model point clouds as reference point clouds.

#### Gingival curve coordinate system adjustment

Through PCA calculation, the positions and main directions of the tooth point cloud and the gingival curve point cloud can be roughly matched. However, the main direction obtained may be incorrect, as shown in Fig. 7a and b. In these cases, subsequent improvement of ICP registration will be senseless. Therefore, dynamic adjustment of the pose is necessary. From the adjusted solution set, suitable initial solutions for improved ICP registration can be selected.

Firstly, the dental model is transformed into the registered coordinate system by using the matrix ${T}_{B}$ obtained above. Then, the AABB tree is constructed for the dental model, and each point in the point cloud *A* is traversed. The nearest point of the AABB tree is searched and the Euclidean distance between the two points is calculated, and the maximum distance between each gingival curve point and the dental model is selected as *L*.

Finally, the dynamic judgment is carried out on the gingival curve cloud. If *L* is larger than threshold value *D*, it means that two point clouds are in the opposite direction of the x-axis or y-axis. Here, point cloud *A* needs to be rotated around x-axis, y-axis and z-axis 180°, to obtain possible correct initial states according to Eq. (8), Eq. (9) and Eq. (10).

8 $$A^{\prime} = \left[ {\begin{array}{*{20}c} 1 & 0 & 0 \\ 0 & { - 1} & 0 \\ 0 & 0 & { - 1} \\ \end{array} } \right]A$$

9 $$A^{\prime} = \left[ {\begin{array}{*{20}c} { - 1} & 0 & 0 \\ 0 & 1 & 0 \\ 0 & 0 & { - 1} \\ \end{array} } \right]A$$

10 $$A^{\prime} = \left[ {\begin{array}{*{20}c} { - 1} & 0 & 0 \\ 0 & { - 1} & 0 \\ 0 & 0 & 1 \\ \end{array} } \right]A$$

Containing the original solution, 4 initial solutions are obtained. The threshold *D* is the practical engineering experience value. If the maximum distance *L* is smaller than *D*, this solution should be collected for subsequent improved ICP registration.

### Improved ICP registration algorithm

**Figure 8:**
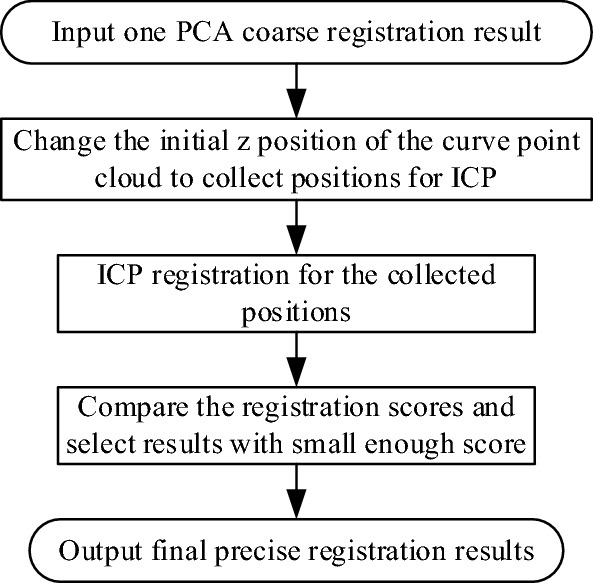
Dynamic hierarchical registration algorithm flow chart.

The results of the previous PCA coarse registration will be used for the ICP fine registration. The improved ICP algorithm based on two point clouds is as follows: change the initial position of the point cloud *A* of the gingival curve to be registered to avoid falling into the local optimal solution. Through the translation registration analysis, it is known that the position change of the xy-axis direction has little effect on the subsequent ICP fine registration results. Therefore, we focus on the z-axis directions adjustment, The specific process is shown in Fig. 8.

- The gingival point cloud *A* is translated along the positive and negative direction of the z-axis of the dental model coordinate system, and the point cloud *A* after each translation is obtained according to Eq. (11).
  11 $$A^{\prime} = \left[ {\begin{array}{*{20}c} {\begin{array}{*{20}c} 1 & 0 & 0 \\ 0 & 1 & 0 \\ 0 & 0 & 1 \\ \end{array} } & {\begin{array}{*{20}c} 0 \\ 0 \\ {dz} \\ \end{array} } \\ {\begin{array}{*{20}c} 0 & 0 & 0 \\ \end{array} } & 1 \\ \end{array} } \right]A$$
- Use the translated point cloud *A* as the point cloud to be registered, and the dental model point cloud *B* as the target point cloud for ICP precision registration.
- Obtain the registration result point cloud, calculate the registration score, which is the closest distance between each point in the result point cloud and point cloud *B*, and obtain the average distance and maximum distance as the ICP fine registration score after each z-layer translation.
- Loop through all translations.

This algorithm solves the problem that the traditional ICP algorithm is inefficient and easy to fall into the local optimal solution.

### Primary calculation experiment

This summary is mainly to verify the feasibility of the above algorithm flow. In this verification, quantity of dental model points is 152,888, and quantity of gingival curve points is 307.

**Figure 9:**
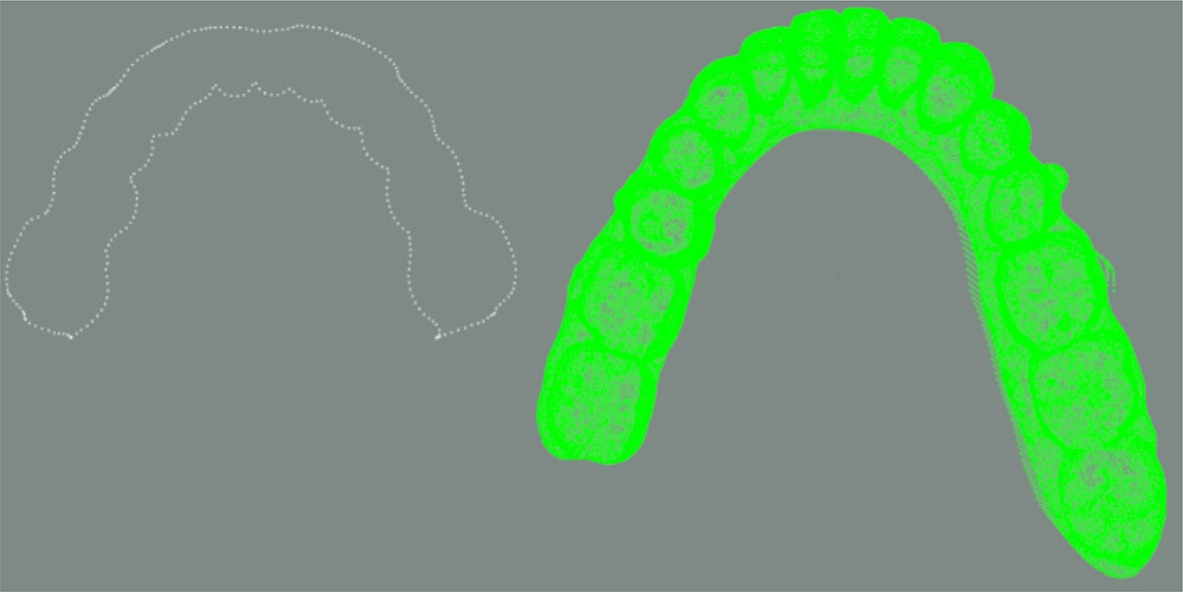
Initial pose.

**Figure 10:**
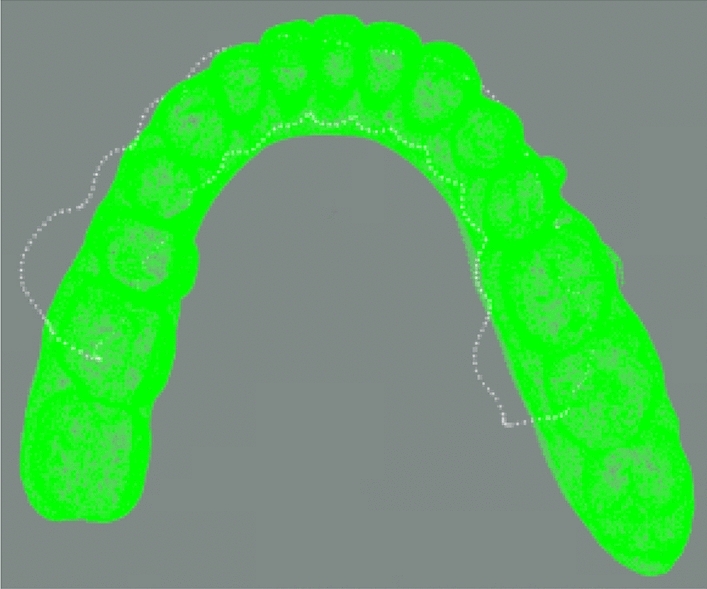
Preliminary registration result.

Firstly, the point cloud data of the dental model is preprocessed by voxel downsampling to reduce the amount of data. Then, the PCA algorithm is used on the two point clouds to align the coordinate systems of two point clouds. The initial pose and one initial state for fine registration are obtained as shown in Figs. 9 and 10. After PCA aligning, the distance between two point clouds are calculated and the results are shown in Table 1.

**Table 1:** Preliminary registration result table (unit: mm).

Minimum distance	0.0119
Maximum distance	5.9287
Average distance	0.0601

**Figure 11:**
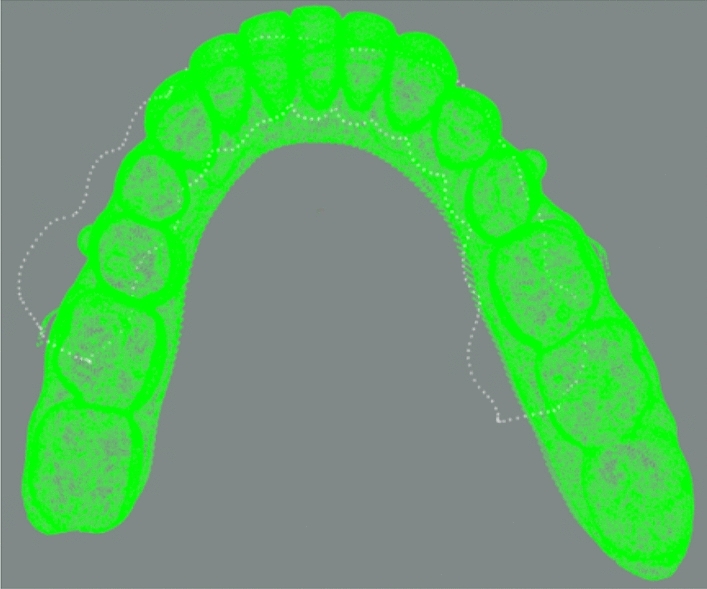
The second case.

The other initial state rotating around y-axis is carried out shown in Fig. 11 and distance in this state is calculated. Distances of two initial states are compared in Table 2. Here, to improve algorithm efficiency, only one initial state is selected priority for z-layer adjustment fine registration. The selection process is to compare the average distances. So the First state is selected in the final improved ICP fine registration. Here, the Z-axis moving range is from -7.5 to 7.5 mm.

**Table 2:** Two initial states when rotate around y-axis (unit: mm).

First		Second	
Minimum distance	0.0119	Minimum distance	0.0332
Maximum distance	5.9287	Maximum distance	5.4946
Average distance	0.0601	Average distance	1.22372

**Figure 12:**
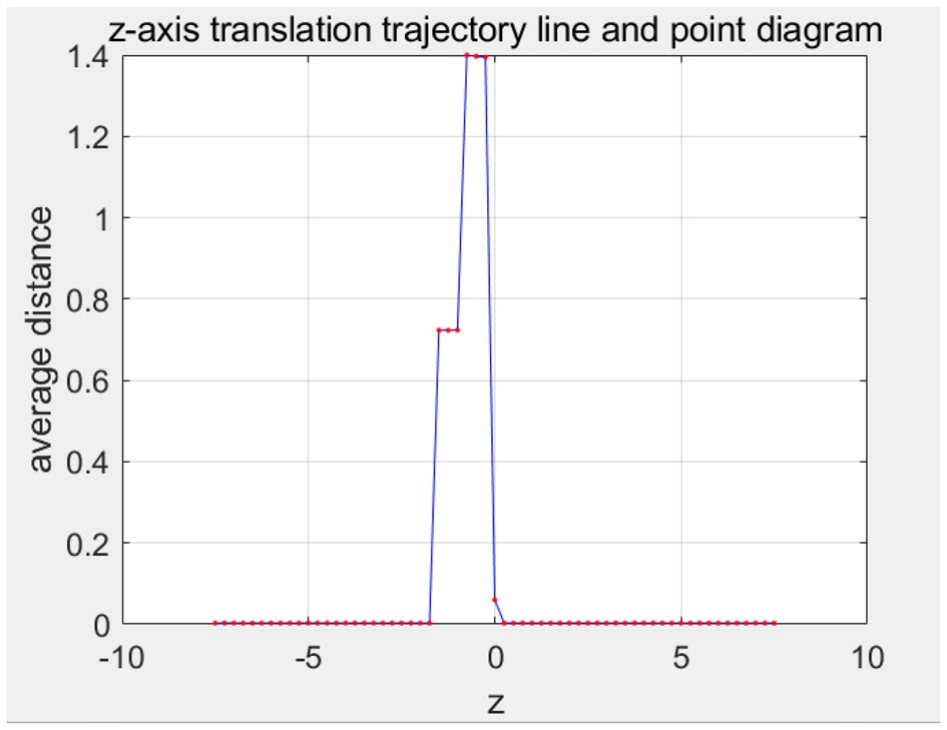
ICP registration average distance when z-axis moving.

In the selected initial state, the PCA-aligned gingival curve is translated along the positive and negative directions of the z-axis, and each translation result is used as the initial point cloud to be registered for ICP fine registration. Then, ICP fine registration is performed by combining the dynamic matching point pair method, and the registration score is calculated. The average distance of final registration result along different z-layer is in Fig. 12.

The number of z-axis translation is given 60. Each translation distance is 0.25 mm. The number of ICP registration of each z position is given 60.

**Figure 13:**
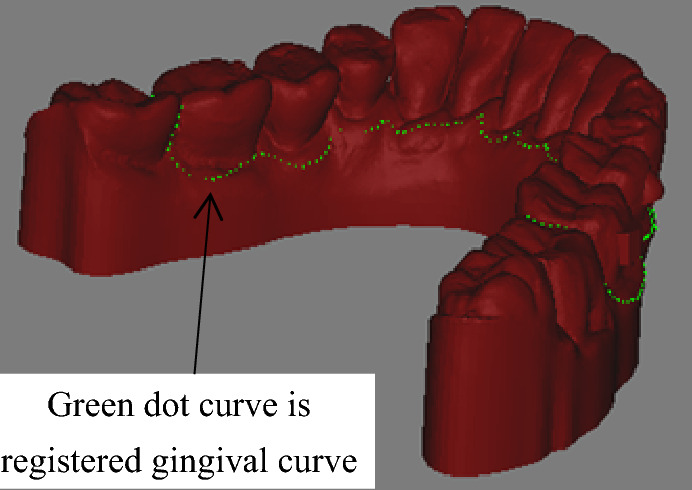
The overall result.

It can be seen from Fig. 12 that if no translation is carried out to change the initial position, the distance is still large and not ideal. However, a better initial position can be obtained by dynamic translation of the z-axis and the final average distance can be improved. As show in Table 3, the average distance is different using varying z-layer registration. The best average distance using the improved dynamic hierarchical registration algorithm based on ICP is 0.0028 mm, and the related maximum distance is 0.03 mm. This result can meet the accuracy requirements in clear aligner robot machining. The final fine registration result is in Fig. 13. The whole calculation time is 2.1 s.

**Table 3:** Fine registration result data table (unit: mm).

Minimum distance	1.2422e−5
Maximum distance	0.0300
Average distance	0.0028

### Conclusion

In this Section, the PCA-ICP registration algorithm is proposed to solve the mismatch problem between dental model point cloud and gingival curve cloud. The initial states searching algorithm based on PCA and the dynamic hierarchical registration algorithm based on ICP improvement are theoretically analyzed, and experiments are designed to verify primarily, which provides a suitable registration result for subsequent machining process. The main research contents of this section are as follows

1. Based on the improved coarse registration algorithm of PCA, the defects left by the traditional PCA algorithm are analyzed. Firstly, the distance threshold is selected, and then the combination structure mode of dynamic matching point pair strategy and ICP coarse registration is used to predict and filter the orientation dynamically. Finally, a more accurate coarse registration result is obtained.
2. Based on the ICP improved dynamic hierarchical registration algorithm, the results are obtained according to the coarse registration algorithm. By changing the position of the coarse registration results, the local optimal solution defect problem is avoided and the accurate registration results are obtained.
3. One case is calculated and the correctness is verified primarily.

## Algorithm validation and processing experiment

This section will analyze and validate the PCA-ICP registration algorithm through more detailed calculation cases experiments. The changes at different stages will be explored and analyzed in different experiments. The visualization software is Cloud Compare v2.10, which can fully verify the effectiveness of the proposed algorithm. Meanwhile, based on the registration results, a suitable tool path will be calculated, and the product will be successfully extracted through a robot flexible cutting case, verifying the effectiveness of the proposed research.In this section, a new dental model is selected with point number 209705, and gingival curve point number is 826.

In order to evaluate the accuracy of the experimental results, this paper uses the average distance between the matched points after registration as the score of the point clouds registration accuracy. In addition, the distance threshold *D* is 14mm.

### Experiment 1: Improved coarse registration experiment based on PCA

In Experiment 1, some different branches in PCA process will be executed and analyzed in detail.

The 4 initial states after PCA analysis alignment are observed and analyzed.

**Figure 14:**
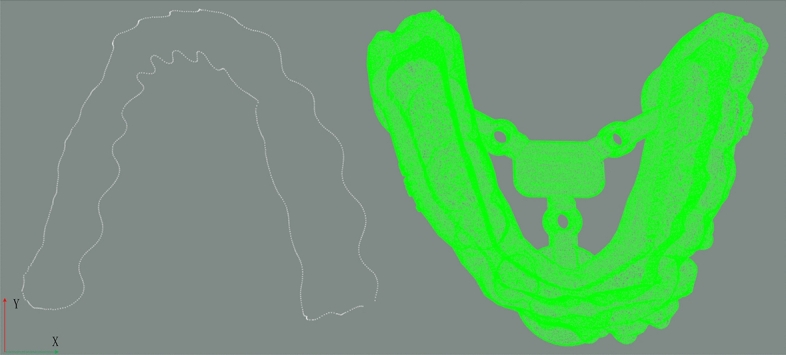
Initial posture relationship.

The input pose Fig. 14 is in accordance with the right-handed coordinate system.

**Figure 15:**
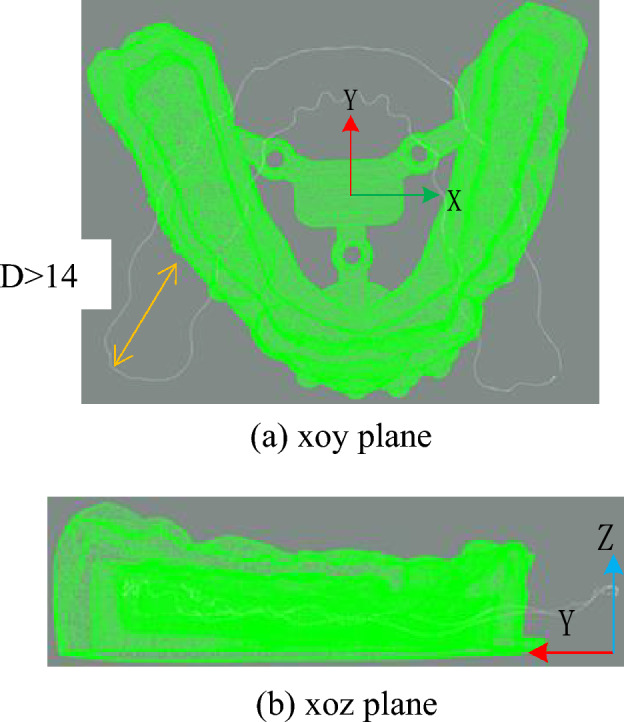
PCA-aligned result.

The two point clouds are aligned using PCA algorithm, and the results are in Fig. 15.

It can be seen that in this two initial states, some points on the gingival curve point cloud are far away from the model point cloud in this case. The maximum distance between one of the gingival curve points and the model point cloud is used to determine whether this initial state is senseful for subsequent fine registration. The judgement is whether the maximum distance is larger than given threshold distance *D*.

Using distance as a condition, these two initial states in y-axis rotation can be easily decided to forsake. The gingival curve point cloud should be rotate 180° around the x-axis to have a new trying job. Table 4 is the comparison of data before and after changing the y-axis direction.

**Table 4:** Variable y-axis direction data comparison table (unit: mm).

Non-adjust		y-axis	
Minimum distance	0.0147	Minimum distance	0.0093
Maximum distance	16.7139	Maximum distance	3.3584

**Figure 16:**
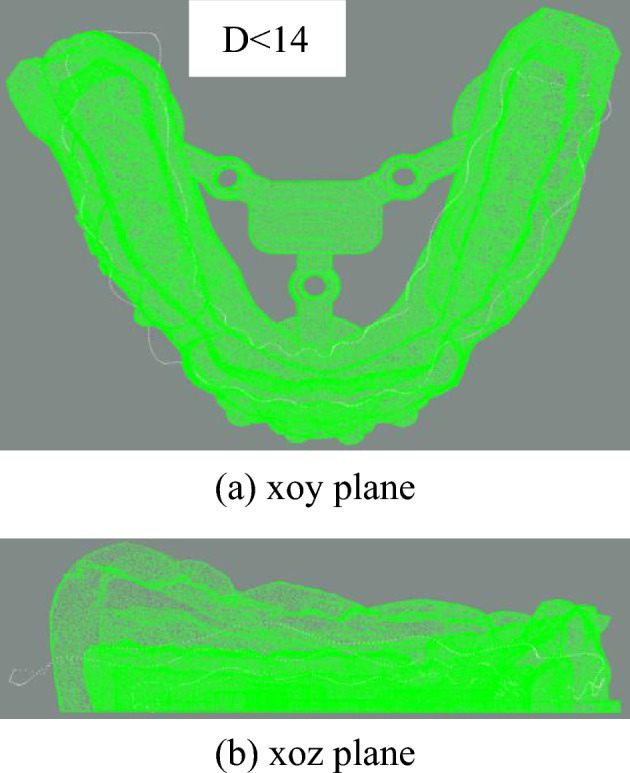
Point cloud transformation y-axis alignment result.

**Figure 17:**
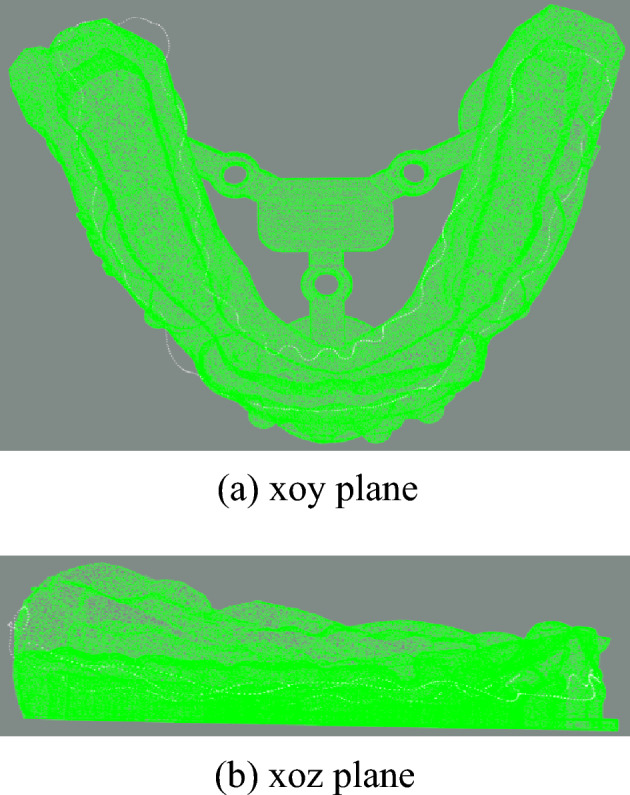
Rotation of the point cloud around the y-axis.

After rotating around x-axis, the rest two initial states point cloud is shown in Figs. 16 and 17.

After rotating around x-axis, the gingival curve point cloud is closer to the dental model, and the unique correct posture cannot be selected by threshold distance directly. Therefore, these two cases rotating around x-axis should be calculated. Figure 17 is another case in which the gingival curve point cloud rotates 180° around the y-axis and 180° around x-axis, which is equivalent as 180° around z-axis.

How can we select the correct initial state of these two cases? Using the idea in Section "Improved ICP registration algorithm", one-z-layer ICP is used to check the distance. The number of ICP registration iterations is 60. Here, the specific data for just one z-layer are given as Table 5.

**Table 5:** Data comparison table of two cases of x axis rotating (unit: mm).

x-axis		z-axis	
Minimum distance	0.0093	Minimum distance	0.0214
Maximum distance	3.3583	Maximum distance	2.8119
Average distance	0.4892	Average distance	0.3682

**Figure 18:**
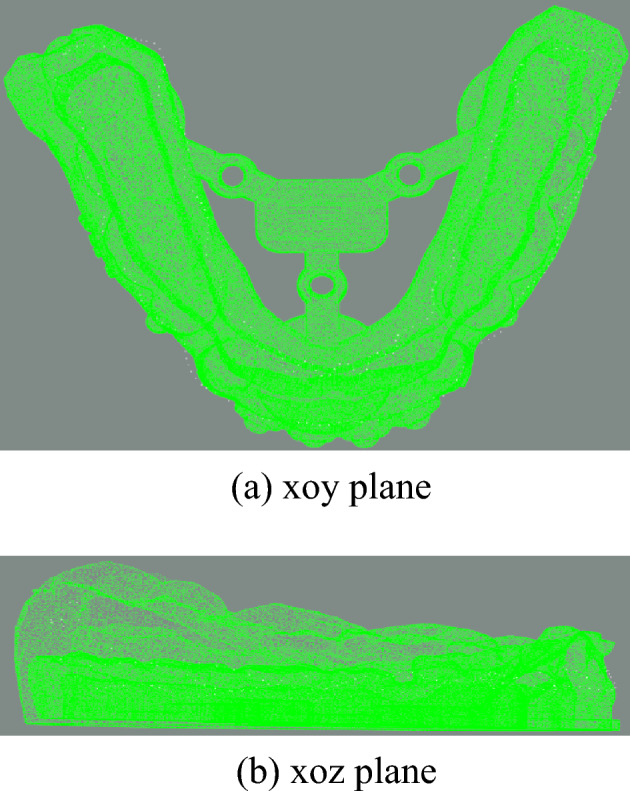
Rotation of the point cloud around the z-axis.

It can be seen that the minimum average distance is 0.3682 mm, and the min–max distance is 2.8119 mm, which illustrates that using one z-layer is not enough. But the improved ICP algorithm can be used in z-axis rotation initial state priority. The result rotating around z-axis is shown in Fig. 18.

Here, if z-axis rotation state is not suitable, that is, using multi-z-layer fine registration is failed to find a low maximum distance, the x-axis rotation state should be tried to find the final suitable result.

### Experiment 2: Analysis of xy-moving translation test

Missing features on the surface, in improved ICP fine registration, variation of position and orientation will lead to different registration result. If all the variations, including three directions rotation and translation, are used for search a satisfied fine registratioin result, the calcuation burden will be huge. So to reduce unnecessary searching job can be analyzed and tried. Based on the huge calculation experiments, the PCA-aligned algorithm can confirm the gingival curve rotation primarily, so the rotation need not adjust. However, the position may not good after PCA-aligned. In different z-layer, the gingival curve may get different result, but some are local optimal. Meanwhile, in the same z-layer, different xy-positions will barely affect the fine registration result.

In this section, continuing with the same case in Experiment 1, xy-moving translation test is carried out. This experiment verifies whether the change of the initial position in the x and y directions has a positive effect on the subsequent registration, paying special attention to the x and y directions as the reference model coordinate system. Specific as follows;

Translation distance step is 2 mm. ICP registration iteration number is 60.

It can be seen from the Table 6 that the average distance after ICP at different initial positions on the xy-axis and the coarse registration result (0.3682 mm) have not large change. Therefore, changing the position of the coarse registration result on the xy-axis has little effect on the subsequent fine registration.

**Table 6:** Mobile location and ICP score table (unit: mm).

location	Average distance	Location	Average distance
(2,0)	0.365737	(0,2)	0.365737
(4,0)	0.495049	(0,4)	0.495049
(-2,0)	0.361668	(0,− 2)	0.361668
(-4,0)	0.377725	(0,− 4)	0.377725
(2,2)	0.494647	(2,− 2)	0.340793
(4,4)	0.493836	(4,− 4)	0.559395
(-2,-2)	0.360675	(− 2,2)	0.377753
(-4,-4)	0.361709	(− 4,4)	0.593385

### Experiment 3: Improved-ICP based on adjusting z-layer

This experiment mainly proves that it can avoid ICP falling into the local optimal solution by changing the position of the initial registration result in the z-axis direction.

The number of z-axis translation is given 60. Each translation distance is 0.25 mm. The number of ICP registration of each z position is given 60.

**Figure 19:**
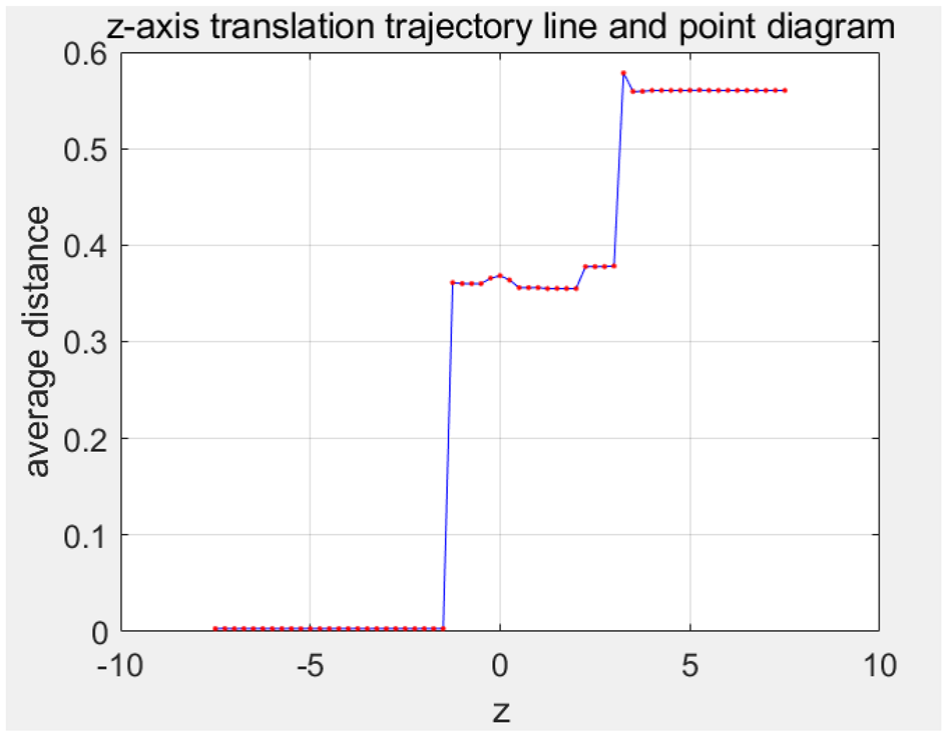
z-axis translation curve.

According to the Fig. 19, we can observe that when moving in two directions along the z-axis, the average distance of the registration result does not change much when the initial moving distance is small. However, as the moving distance increases, we can observe the emergence of two trends.

First of all, when the square moves up, the score shows a gradually increasing trend, which means that the registration result becomes worse after the square shift up the z-axis. This may indicate that the translation results in greater error or reduced alignment.

Secondly, when the negative side moves upward, the score decreases gradually, which indicates that the registration result becomes better. This may indicate that translations along the negative z-axis can improve alignment accuracy and consistency.

Finally, at a suitable point, we can observe that the registration result converges to a good value, such as Table 7, where the score is the smallest, 0.00299027 mm. This means that there is an optimal translation matching position in both the positive direction and the negative direction, and the best registration result can be obtained, which solves the problem of local optimal solution caused by poor coarse registration position.

**Table 7:** Fine registration result data table (unit: mm).

Minimum distance	2.0838e−5
Maximum distance	0.03296
Average distance	0.0030

**Figure 20:**
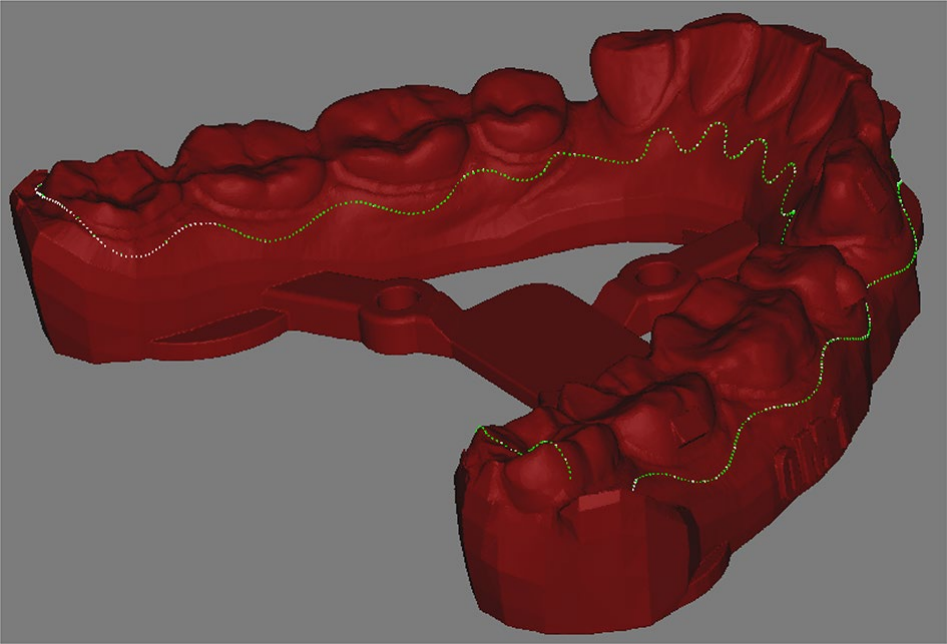
The final registration result.

The aligned data import software (cloud comparison v2.10) is compared with the standard results provided by third-party data, as shown in Fig. 20, white gingival curve is the standard result, and green is the registration result of the algorithm in this paper. The whole calculation time is 5.8 s.

To ensure registration stability, quantities of cases are calculated, including different cases and different orientations of point cloud pairs. With enough rational initial cases are found for ICP registration, all the cases can be calculated successfully. The PCA method can basically ensure posture relationship between two point clouds. The z-layer dynamic hierarchical is to ensure to find the best registration result. Other factors can be ignored for large quantities. Surely, for some extremely case, when no correct result is found, some searching factors have to be open, which will lead to much more calculation times.

### Tool path generation and processing experiment

#### Tool path generation

Before working, an interference-free milling tool path should be generated. Generally, for traditional tool path generation of milling, it is enough to represent the position and orientation of tools with a point on the tool axis and a tool axis direction because these tools can be looked as surfaces of revolution^20,21^. Without loss of generality, let’s take 6-axis milling for example, the milling cutter can retract along the tool axis direction to avoid interference. Usually, C space approach was used to generate interference-free tool path automatically^22,23^.

it is difficult to generate interference-free tool path automatically due to its complexity of Teeth shape. Therefore, current tool path generation methods will fail in this situation and it is necessary to develop new method to generate tool path for flexible milling.

Therefore, the detailed calculation can be described as follows. TCP posture $\zeta^{t} = \left[ {p^{t} ;e_{1}^{t} ,e_{2}^{t} ,e_{3}^{t} } \right]$ is used to represent the positions and postures of the Milling cutter. $p^{t}$ denotes the position vector. $e_{1}^{t}$ is unit vector and parallel to the axis of milling tool. $e_{2}^{t}$ is unit vector which is the x-axis of the tool. $e_{3}^{t}$ is vertical to $e_{1}^{t}$ and $e_{2}^{t}$, $\zeta^{h} = \left[ {p^{h} ;e_{1}^{h} ,e_{2}^{h} ,e_{3}^{h} } \right]$ denotes the position and direction of the point to mill, $e_{1}^{h}$ Indicates the normal direction of a point, $e_{1}^{h}$ and $e_{2}^{h}$ determine the work plane. To simplify the calculation, let $e_{1}^{t} = e_{1}^{h}$, $e_{2}^{t} = e_{2}^{h}$ during the process of tool path generation.

Therefore, if $p^{t}$ is calculated, the tool position can be achieved. Since $p^{t}$ is within the work plane, it can be expressed as $p^{t} = p^{h} + xe_{1}^{h} + Ye_{2}^{h}$.

Since $\zeta^{h} = \left[ {p^{h} ;e_{1}^{h} ,e_{2}^{h} ,e_{3}^{h} } \right]$ is known for a given part, a series of tool positions and postures of milling tool can be described as $\left[ {p^{h} + x_{i} e_{1}^{h} + y_{i} e_{2}^{h} ; e_{1}^{h} ,e_{2}^{h} ,e_{3}^{h} } \right]$.

Finally, according to the programming language of ABB robot, a post-processing program is written using C +  + to generate robot processing program.

**Figure 21:**
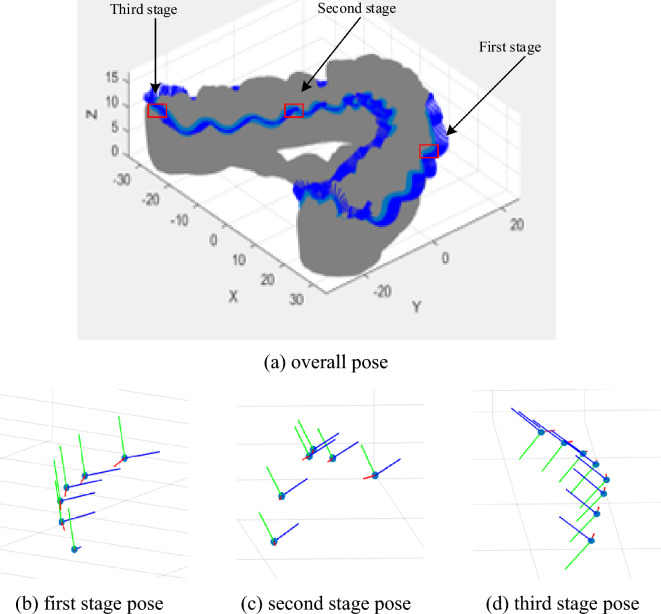
Overall path and local enlarged orientation.

Based on this orientation calculation process, orientation sequence on the gingival curve of one dental model is calculated as shown in Fig. 21, where red is the x-axis, green is the y-axis, and blue is the z-axis.

#### Clear aligner flexible processing experiment

**Figure 22:**
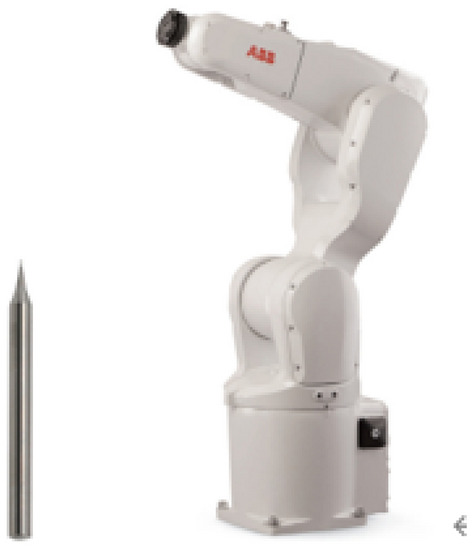
Sketch of cutting tools and industrial robots.

In order to verify the processing effect, tungsten steel single-edge flat-bottom milling cutter and ABB industrial six-axis robot Fig. 22 are used for processing. The core technology of ABB robot is motion control system, which has the advantages of high precision and high reliability.

**Figure 23:**
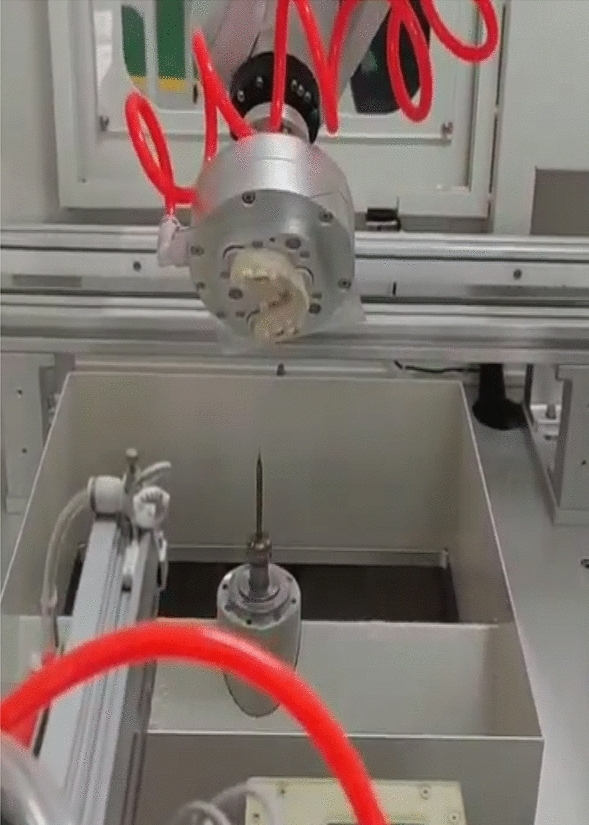
Installation location.

The installation of the tool and the robot is in Fig. 23. In the process of machining, the tool only does rotary motion. The end of the robot does feed movement.

The tool path file is imported into the robot program. The joint planning of the robot adopts the algorithm provided by the manufacturer. The tool spindle speed is 35,000 r / min, the cutting depth is 2 mm, and the average feed speed is 1320 mm / min.

**Figure 24:**
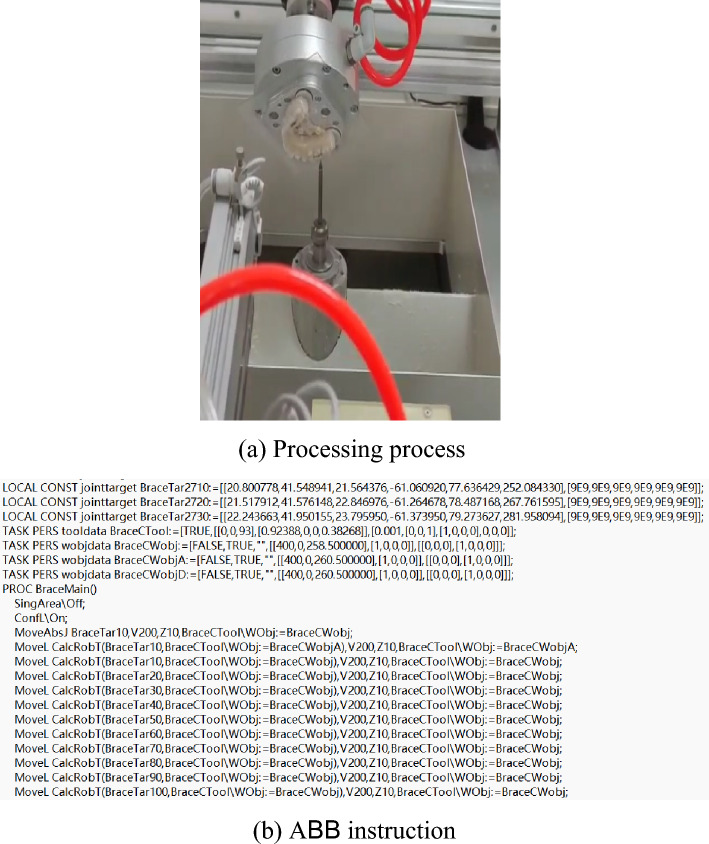
Robot machining process.

It takes about 15 s to cut a circle during processing. The machining process is in Fig. 24.

The processing time of this case is 15 s, the manual cutting time is 70 s, and the manual polishing time is 65 s. The average efficiency of robot processing is 20 s / piece, and the average efficiency of manual production is 120 s / piece. Compared with manual processing, robot processing has the advantages of high efficiency, path accuracy and surface smoothness. As shown in Figs. 10 and 25 in Ref.^8^.

**Figure 25:**
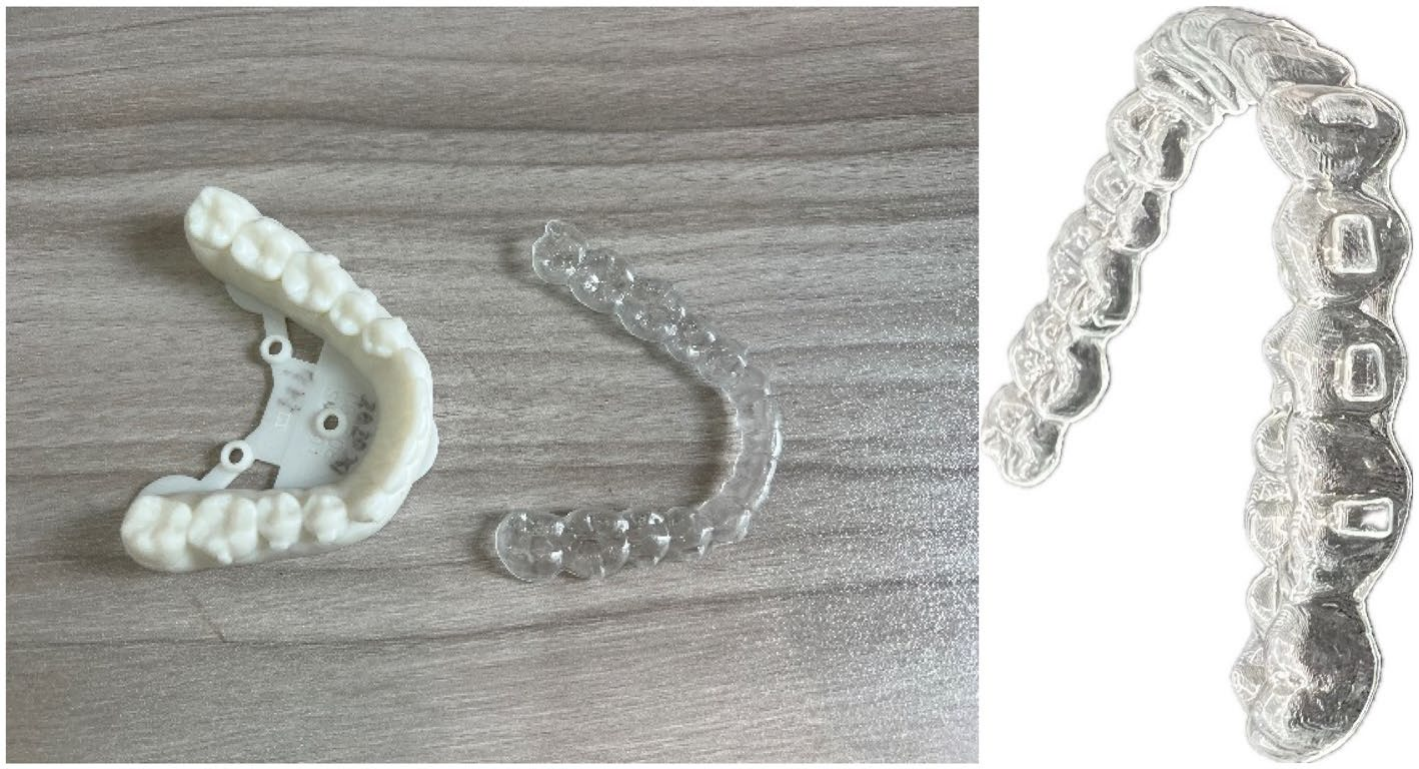
Finished clear aligner product.

### Conclusion

This section analyzed and discussed the problems encountered in the registration process, and process the clear aligner using ABB industrial robot by generating intereference-free smooth tool path.Experiment one mainly analyzes and discusses the coordinate axis reverse problem that may occur when the traditional PCA principal component analysis method processes the point cloud data. A method of using a unified local coordinate system is proposed to quickly deal with the reverse situation on the z-axis. In addition, by using the maximum distance between two point clouds as a condition, it is possible to accurately and quickly determine whether there is a reverse situation on the y-axis. Finally, for the situation on the x-axis, ICP coarse registration is used for processing, and the registration score is calculated. By comparing the size of the score, the correct results are selected to complete the coarse registration. Experiment two and three are to solve the problem that the ICP fine registration fall into the local optimal solution. In Experiment two, The suitable xy-position of gingival curve is tried to find. The purpose is to verify whether the ICP can avoid falling into the local optimal solution. The experimental results show that xy-position is senseless for the success rate of the ICP. In Experiment three, aiming at the problem that the ICP fine registration stage is easy to fall into the local optimal solution, the problem is solved by changing the position of the point cloud to be registered in the z-axis direction. The experimental results show that this method can effectively avoid ICP falling into local optimal solution. This three calculation experiments have analyzed the robustness of the PCA-ICP algorithm.

The machining experiment is carried out to validate the registration result. The milling posture is calculated based on the well-registered curve. The ABB post-processed code sequence is generated based on the milling posture. The clear aligner is machined successfully with enough accuracy and surface smoothness.

## Summary and future work

### Summary

This article proposes solutions to two key issues in the manufacuting of clear aligners. One key issue is the mismatch between the given gingival curve and the dental CAD model, and the other issue is the planning of smooth and interference-free robot cutting path based on the gingival curve.

To address the problem of inaccurate matching between the gingival curve and the dental CAD model, the PCA-ICP registration algorithm is proposed. The PCA algorithm is used to initially match the gingival curve and the dental CAD model. Possible initial posetures of the gingival curve that are suitable for subsequent fine matching are calculated. Since the gingival curve and the dental model features cannot be directly matched, an improved ICP algorithm is proposed. Based on the analysis of the matching efficiency, a z-layer dynamic hierarchical approach is applied. The ICP registration is applied to different layers. The accuracy is validated using maximum distance error and average distance error of two point clouds. Simulation calculation analysis shows that the maximum deviation of point cloud registration is 0.03 mm, which meets the machining error requirements of the aligners. Therefore, it can be concluded that the PCA-ICP algorithm is suitable for matching the gingival curve and the dental model.

Finally, the calculated pose sequence is sent to an ABB robot for inverse kinematics, obtaining the machining path. Experimental machining of the dental model shows that the cut aligners are removable, indicating that the machining error meets the requirements. This proves that the algorithm proposed in this article can successfully complete the cutting of aligners.The PCA-ICP algorithm can solve the coordinate system mismatch between two point clouds, which one cloud is curve cloud, lacking of surface features. This proposed algorithm may expand the registration algorithms. More types of point clouds matching problems can be solved based on this algorithm. The point cloud pairs which are part and whole can be matched using surface feature recognizing. But if the “part” cloud is lack of surface feature, the proposed method can also help to find the correct posture to match the whole cloud. In clear aligner manufacturing problem, the gingival curve and dental model generations are in designing and manufacturing environments. The proposed algorithm can automatically connect the two different environments. These two clouds are brought together into the same environment.

### Future work

However, there are still some points to be optimized in this algorithm, especially in registration process. For example, to select suitable initial states for improve-ICP registration quickly, the ICP is used regardless of inaccuracy after PCA alignment. Can this operation accurately select the correct initial state or can other methods achieve this goal? More different cases should be tested deeply. In the other hand, we have proved experimentally that xy-axis direction varying has little impact for ICP result. But the z-layer varying will decide the ICP success rate. How to select z-layer but not traverse, or using a suitable z-axis moving step to traverse, is another question.

In the future, the ICP registration results will collect and the deep learning method is allowing to be used to quickly and accurately select an input to decrease ICP registration times, including initial state selection, a suitable z-layer position calculation or a moving step confirmation.

### Supplementary Information

Supplementary Information 1.

Supplementary Information 2.

Supplementary Information 3.

Supplementary Information 4.

Supplementary Information 5.

Supplementary Information 6.

## Data Availability

The datasets generated and analysed during the current study are not publicly available due to the data ownership belonging to Dental Hospital but are available from the corresponding author on reasonable request.
